# Microenvironment-Driven Reprogramming in Colorectal Cancer Liver Metastasis: Metabolic, Phenotypic, and Immune Adaptation

**DOI:** 10.3390/ijms27146206

**Published:** 2026-07-11

**Authors:** Xiaoli Mu, Wenjun Meng, Lingnan Zheng

**Affiliations:** 1 Health Management Center, General Practice Medical Center, West China Hospital, Sichuan University, Chengdu 610041, China; mxl0910@scu.edu.cn; 2 Department of Pain Management, West China Hospital, Sichuan University, Chengdu 610041, China; mwj1995@scu.edu.cn; 3 Department of Oncology, West China School of Public Health and West China Fourth Hospital, Sichuan University, Chengdu 610041, China

**Keywords:** colorectal cancer, liver metastasis, tumor microenvironment, metabolic reprogramming, epithelial–mesenchymal plasticity, immune evasion

## Abstract

Liver metastasis is a major cause of mortality in patients with metastatic colorectal cancer and reflects the selective pressures imposed by the hepatic niche. This review summarizes how the liver microenvironment may reshape disseminated colorectal cancer cells through three interconnected programs: metabolic reprogramming, phenotypic plasticity, and immune evasion. Metabolically, metastatic cells adapt to the glucose-poor and lipid-rich hepatic milieu by switching between glycolysis and oxidative phosphorylation, activating gluconeogenesis, increasing glutamine dependence, and remodeling lipid utilization. Phenotypically, stromal cues such as TGF-β and HGF may promote epithelial–mesenchymal plasticity, thereby supporting invasion, survival, and metastatic outgrowth. Immunologically, the hepatic niche facilitates immune escape through PD-L1 upregulation and the recruitment or polarization of suppressive myeloid and regulatory T-cell populations. We further discuss therapeutic opportunities arising from these vulnerabilities, including inhibition of metabolic dependencies, blockade of TGF-β/FAK-driven plasticity, and combination immunotherapy targeting the PD-1/PD-L1 axis together with the liver immune microenvironment. Finally, we highlight the need for biomarker-guided patient stratification, more faithful preclinical models, and rational combination strategies to overcome adaptive resistance and improve outcomes in colorectal liver metastasis.

## 1. Introduction

Colorectal cancer (CRC) is the third most commonly diagnosed malignancy and the second leading cause of cancer-related mortality worldwide [1,2]. Among patients with metastatic CRC, the liver is one of the most prevalent and clinically consequential metastatic sites, affecting approximately 50–60% of individuals during disease progression. Once liver metastasis is established, prognosis worsens substantially, with the 5-year survival rate falling below 15% for unresectable disease [3,4]. Despite advances in systemic therapies, including combination chemotherapy, anti-EGFR/anti-VEGF targeted agents, and immune checkpoint inhibitors (ICIs), durable remission is rarely achieved in established liver metastases. This highlights an urgent need to dissect hepatic microenvironment-driven biological mechanisms underlying metastatic seeding and therapeutic resistance. Although both the liver and lung are frequent metastatic destinations in colorectal cancer, they impose biologically distinct selective pressures on disseminated tumor cells [5,6]. Liver colonization is facilitated by portal venous drainage from the intestine and occurs within a sinusoidal vascular bed enriched in Kupffer cells (KCs), liver sinusoidal endothelial cells (LSECs), hepatic stellate cells (HSCs), hepatocytes, bile acids, and dynamic nutrient and oxygen gradients [7]. This hepatic environment may create a metabolically active and immunotolerant niche that favors glucose, lipid, and amino acid rewiring, myeloid-cell-mediated immune suppression, and reduced responsiveness to immune checkpoint blockade [8]. By contrast, lung metastasis develops in an aerated alveolar-capillary niche shaped by pulmonary epithelial cells, alveolar macrophages, fibroblasts, surfactant-associated signals, and high oxygen exposure, where metastatic adaptation is more closely linked to vascular arrest, alveolar inflammation, extracellular matrix (ECM) remodeling, and pulmonary immune surveillance [5,9]. Thus, liver metastasis should be considered not only an anatomical consequence of tumor dissemination but also an organ-specific reprogramming process distinct from lung metastasis.

As the principal metabolic organ, the liver receives dual blood supply from the portal vein and hepatic artery and contains abundant non-parenchymal cells, including KCs, LSECs, and HSCs [10]. These physiological features may contribute to an immunosuppressive and pro-angiogenic pre-metastatic niche that supports early CRC cell dissemination and colonization. Following successful hepatic engraftment, metastatic cancer cells encounter a microenvironment fundamentally distinct from the primary tumor, characterized by glucose depletion, excessive lactate efflux, and altered lipid homeostasis. Tumor proliferation can further aggravate metabolic imbalance by exacerbating glucose deprivation and lactate accumulation, thereby supporting malignant progression [9]. In this environment, cancer cells may undergo adaptive reprogramming in response to local microenvironmental cues, with cellular states being remodeled to acquire metabolic flexibility, evade hepatic immune surveillance, and develop therapeutic resistance [11].

Existing reviews have largely discussed metabolic, phenotypic, or immune alterations in isolation. However, in colorectal liver metastasis (CRLM), these processes are unlikely to operate independently; instead, they appear to arise from hepatic niche-driven selective pressures and reciprocal signaling among cancer cells, stromal cells, and immune cells. Recent advances in single-cell sequencing and spatial multi-omics have provided new mechanistic insights into these adaptive reprogramming events [12,13]. Here, we propose that CRLM is driven by a hepatic niche-dependent reprogramming program integrating metabolic adaptation, phenotypic plasticity, and immune escape. We therefore summarize the molecular logic of these interconnected processes and discuss how they may be therapeutically exploited to overcome adaptive resistance.

## 2. The Liver Niche as a Driver of Reprogramming

The liver microenvironment provides selective pressures that may promote the reprogramming of metastatic CRC cells. Unlike most peripheral tissues, the liver receives dual blood supply from the hepatic artery and portal vein, creating distinct oxygen and nutrient gradients within the hepatic lobules [14,15]. This blood-flow pattern, together with the liver’s role as a metabolic hub, creates a microenvironment that differs substantially from that of the primary colorectal tumor.

**Figure 1:**
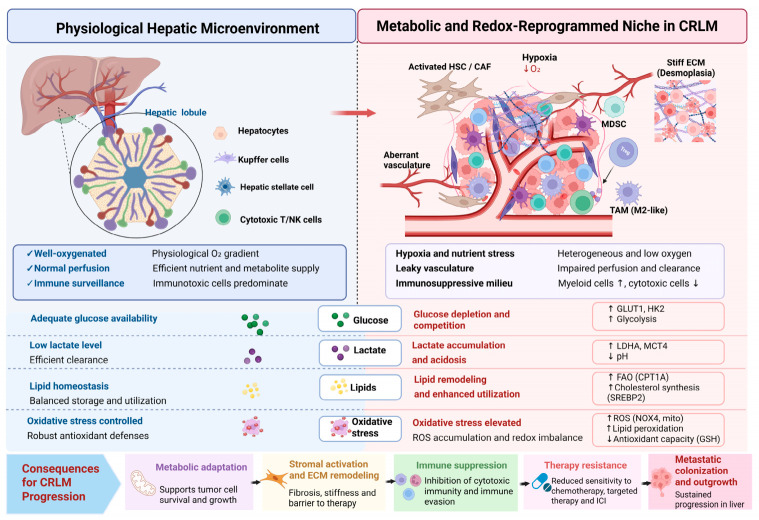
Metabolic and redox remodeling of the hepatic microenvironment during colorectal liver metastasis. This schematic compares the physiological hepatic microenvironment with the metabolic and redox-reprogrammed niche in colorectal liver metastasis (CRLM). In the normal liver, hepatocytes, quiescent hepatic stellate cells (HSCs), Kupffer cells, liver sinusoidal endothelial cells (LSECs), and cytotoxic T/NK cells are organized within a well-perfused sinusoidal structure that maintains oxygen and nutrient gradients, metabolic homeostasis, and immune surveillance. During CRLM progression, metastatic CRC cells remodel the hepatic niche through hypoxia, aberrant vasculature, extracellular matrix (ECM) deposition, activation of HSCs/cancer-associated fibroblasts (CAFs), and enrichment of immunosuppressive cells, including tumor-associated macrophages (TAMs), myeloid-derived suppressor cells (MDSCs), and regulatory T cells (Tregs). The lower panel highlights the major metabolic and redox alterations, including glucose depletion and competition, lactate accumulation and acidosis, lipid remodeling with enhanced fatty acid oxidation, and elevated oxidative stress with redox imbalance. These changes collectively support metabolic adaptation, stromal activation, immune suppression, therapeutic resistance, and metastatic colonization in the liver.

Within this structural framework, resident non-parenchymal cells cooperatively establish a pro-metastatic milieu. KCs act as immune sentinels under physiological conditions but can adopt immunosuppressive phenotypes in response to tumor- or injury-derived signals, characterized by IL-10 and TGF-β secretion alongside impaired antigen-presenting capacity [16]. Beyond intrinsic microenvironmental cues, clinical chemotherapy further modulates KC polarization. Specifically, chemotherapy-induced reprogramming of liver-resident KCs toward a LEPR^+^ phenotype facilitates immune evasion and chemoresistance development in colorectal liver metastases [17]. In addition to KCs, HSCs represent another resident non-parenchymal cell population that may contribute to the pro-metastatic hepatic microenvironment. HSCs, vitamin A-storing pericytes residing within the space of Disse, remain quiescent under homeostatic conditions. In response to tumor-derived and inflammatory stimuli, HSCs become activated and transdifferentiate into cancer-associated fibroblast (CAF)-like cells, which deposit ECM components and secrete fibrogenic and paracrine factors (e.g., TGF-β, HGF). These HSC-derived CAF-like cells can remodel the ECM and secrete cytokines, thereby contributing to an immunosuppressive tumor microenvironment (TME) that supports metastatic colonization [18]. Concurrently, LSECs participate in angiocrine and immunoregulatory crosstalk within the liver, with PD-L1-driven tolerogenic signaling identified in LSECs under defined experimental contexts [19,20]. Together, interactions among these resident cells and recruited immunosuppressive populations, including MDSCs and Tregs, may create a permissive niche for tumor cell seeding and reprogramming [21] (Figure 1).

In addition to its cellular composition, the liver provides a unique biochemical environment that presents both advantages and disadvantages for metastatic CRC cells. On the one hand, the abundance of nutrients, dense vascular network, and relative immune tolerance facilitate the early survival and establishment of disseminated tumor cells. On the other hand, these cancer cells contend with bile acid toxicity, oxidative stress, fluctuations in oxygen concentration, and intense metabolic competition in the hepatic microenvironment [22]. These stresses may promote rapid metabolic adaptation, including increased reliance on alternative substrates such as fatty acids and glutamine and activation of stress-adaptation pathways. Thus, the liver microenvironment should be viewed not merely as a site of metastatic deposition but also as an active source of selective pressure that shapes tumor cell behavior during hepatic colonization.

Overall, the liver microenvironment helps shape the fate of disseminated tumor cells and provides a framework for understanding the metabolic, phenotypic, and immune reprogramming processes discussed below.

## 3. Metabolic Reprogramming

Metabolic reprogramming enables tumor cells to adapt to the hepatic microenvironment and survive under nutrient limitation, hypoxia, and immune pressure [23]. Coordinated changes in glucose, lipid, and amino acid metabolism appear to support this adaptive process. These metabolic adaptations have been associated with the successful establishment of metastatic cells [24].

### 3.1. The Glycolysis-OXPHOS Switch

During liver metastasis, disseminated CRC cells exhibit significant metabolic plasticity. In the early stages of metastasis, these cells may rely on aerobic glycolysis (i.e., the Warburg effect) to rapidly generate ATP. CMTM6 has been reported to inhibit lysosomal degradation of the glucose transporter GLUT1, thereby sustaining glycolytic flux [25]. Enhanced glycolysis can lead to lactate accumulation and acidification of the local microenvironment. This acidic metabolic state may impair T-cell and NK cell surveillance and favor M2-like macrophage polarization. At the same time, to meet the increasing energy demands, migratory cells also upregulate mitochondrial oxidative phosphorylation (OXPHOS). Mitochondrial MTA1 can bind ATP5A, enhance ATP synthase activity, and activate downstream mTOR signaling [26]. This dual reliance on glycolysis and OXPHOS may support early colonization and may represent a metabolic vulnerability; however, direct clinical validation of this strategy in CRLM remains limited.

### 3.2. Ectopic Gluconeogenesis

CRC cells may adapt to the glucose-deprived hepatic environment not only by altering ATP production but also by activating gluconeogenic programs. In the glucose-deprived liver microenvironment, FOXO1- and CREB-mediated transcriptional programs can induce the expression of the gluconeogenic enzymes PCK1 and G6PC, thereby helping tumor cells maintain metabolic homeostasis and meet biosynthetic demands [27]. These adaptations may allow cancer cells to coordinate substrate uptake, catabolism, and endogenous glucose production within the nutrient-restricted liver, thereby supporting survival and establishment.

### 3.3. Glutamine Dependency

Glutamine is an important carbon and nitrogen source for tumor cells, playing a role in the citric acid cycle, nucleotide and lipid synthesis, and the generation of reducing equivalents. Some aggressive CRC cells exhibit glutamine dependence [28]. SLC1A3, a glutamate transporter, and GLS, a glutaminase, are important components of the glutamine/glutamate metabolic pathway. Increased expression of these molecules has been associated with CRC progression, invasiveness, and poor prognosis, and pathway inhibition has been proposed as a potential anti-metastatic strategy [29].

CRLM-derived cells have been reported to rely on glutamine metabolism, a process that may be regulated by YTHDF1 [30,31]. YTHDF1, an m6A RNA methylation reader, can enhance GID8 translation in an m6A-dependent manner, and increased GID8 expression may promote glutamine metabolism and CRC progression. The YTHDF1–GID8–glutamine metabolism axis has therefore been implicated in CRC progression and may represent a potential metabolic vulnerability [32].

### 3.4. Lipid Metabolism

The lipid-rich liver microenvironment may promote extensive lipid remodeling in disseminated CRC cells. Under glucose-restricted conditions, metastatic cells may enhance fatty acid oxidation (FAO) by upregulating carnitine palmitoyltransferase 1A (CPT1A), the rate-limiting enzyme of FAO, thereby using exogenous lipids as an energy source [33]. Cholesterol synthesis can also be enhanced by microenvironmental signals, such as the HGF-mediated c-MET/PI3K/AKT/mTOR pathway, and this dependence on lipid synthesis can also be targeted, as statins have been shown in preclinical studies to inhibit liver colonization [34]. Systemic metabolic disorders, such as non-alcoholic fatty liver disease (NAFLD), may further amplify lipid-driven metastatic progression. In such cases, fatty liver cells may release extracellular vesicles (EVs) enriched in CYP2E1, a cytochrome P450 enzyme involved in reactive oxygen species generation. When CRC cells take up these vesicles, they are induced to produce oxidative stress, and their oncogenic signaling pathways are further reshaped. These findings suggest that host metabolic dysfunction may contribute to metastatic progression [35].

### 3.5. Gut Microbiota-Derived Metabolites and Hepatic Metabolic Adaptation

The gut–liver axis may further shape metabolic reprogramming during CRLM. Microbial metabolites and products enter the liver through the portal circulation, exposing disseminated CRC cells and hepatic stromal cells to gut-derived signals. Secondary bile acids, short-chain fatty acids, lipopolysaccharide, and other microbial components can influence hepatic lipid metabolism, oxidative stress, inflammatory signaling, and epithelial plasticity. In particular, bile acids modified by intestinal microbiota have been implicated in colorectal tumor growth and immune regulation, while dysbiosis-associated changes in bile acid metabolism may contribute to hepatic niche remodeling [36]. Recent evidence suggests that intratumoral bacteria, such as *Escherichia coli*, may enhance lactate production in CRLM, thereby linking microbial colonization to lactate metabolism, macrophage polarization, and immune suppression [37]. Although the causal contribution of specific microbial species or metabolites to CRLM metabolic dependencies remains incompletely defined, the gut–liver axis provides a plausible route through which intestinal ecology may influence the metabolic state of the liver metastatic niche [38].

## 4. Phenotypic Plasticity: EMT/MET Dynamics

Beyond metabolic adaptation, hepatic colonization may require disseminated CRC cells to engage phenotypic plasticity and transition across epithelial, partial EMT, hybrid epithelial/mesenchymal (E/M), and mesenchymal states rather than undergo a rigid binary switch. EMT is increasingly viewed as a reversible continuum, termed epithelial–mesenchymal plasticity (EMP), which allows tumor cells to adapt to the hepatic niche without irreversible commitment to a mesenchymal state [39].

### 4.1. HSCs as Drivers of EMT

In the hepatic niche, CRC cells encounter activated HSCs that secrete TGF-β and HGF [33]. HGF signaling through c-MET may promote tumor cell survival, motility, and invasive capacity, thereby facilitating metastatic colonization within the liver. Tumor-intrinsic TGF-β signaling can suppress the epithelial marker E-cadherin through canonical transcriptional repressors, including Snail, ZEB1, and Twist, while upregulating mesenchymal markers such as N-cadherin and vimentin. Beyond these canonical EMT regulators, upstream transcription factor 2 (USF2) has been reported to mediate TGF-β-induced EMT in CRC by upregulating the calcium-binding protein S100A8, thereby enhancing metastatic potential [40]. Notably, reciprocal crosstalk may occur between CRC cells and HSCs. Tumor-derived paracrine signals can further reprogram activated HSCs by upregulating GLUT1 expression and enhancing glycolysis, thereby sustaining a pro-tumorigenic CAF-like phenotype. These HSC-derived CAF-like cells may secrete cytokines and matrix components that further support CRLM progression [18].

### 4.2. MET and Metastatic Outgrowth

Once micrometastatic lesions form, disseminated cells often undergo the reverse process of EMT, namely mesenchymal–epithelial transition (MET). This phenotypic reversion can restore epithelial markers such as E-cadherin and enhance cell–cell adhesion, thereby supporting collective proliferation and macroscopic tumor outgrowth [41]. Between fully mesenchymal and fully epithelial states, hybrid E/M cells represent an important subpopulation within this spectrum. These cells retain their adhesive capacity while acquiring survival advantages, thereby promoting the formation of clusters of circulating tumor cells (CTCs) and subsequent liver colonization [42]. Thus, CRC cells may not remain fixed in a single phenotypic state but instead transition dynamically between EMT and MET during CRLM progression.

This EMT-MET plasticity is also mechanistically linked to other adaptive reprogramming processes. For example, TGF-β signaling may coordinate EMT, metabolic remodeling, including enhanced fatty acid oxidation, and immune evasion through PD-L1 upregulation [43,44]. Accordingly, targeting upstream pathways such as TGF-β or HGF, or modulating downstream effectors such as integrins and FAK, may disrupt multiple adaptive programs. From a translational perspective, these approaches may help restrict metastatic plasticity and improve sensitivity to chemotherapy or immunotherapy, although clinical validation in CRLM remains limited.

## 5. Immune Reprogramming

The liver is inherently immunotolerant and is continuously exposed to antigens and microorganisms from the gut [45]. While this characteristic helps prevent excessive inflammation, it also creates a favorable environment for metastatic colonization. CRC cells exploit this tolerant environment by reshaping the liver’s immune state, thereby shifting immune surveillance toward immune evasion.

### 5.1. Kupffer Cell-Mediated Immunosuppression

KCs, the liver-resident macrophages, are among the first immune cells to encounter disseminated CRC cells [16]. Instead of mounting an effective antitumor response, KCs may be polarized by CRC cells toward an M2-like immunosuppressive phenotype. These polarized KCs can secrete IL-10, TGF-β, and IL-6, thereby suppressing cytotoxic T-cell activity, promoting Treg recruitment, and supporting tumor cell survival [46]. Pharmacological inhibition of colony-stimulating factor 1 receptor (CSF1R), a pathway involved in Kupffer cell survival and polarization, has shown activity in preclinical models of CRLM [47].

### 5.2. MDSC, TAM, and Treg Recruitment

Tumor colonization can induce the release of inflammatory mediators, including CCL2, CCL5, and CXCL5, which recruit MDSCs, tumor-associated macrophages (TAMs), and Tregs into the hepatic niche and contribute to an immunosuppressive microenvironment [48,49]. These recruited populations can further reinforce hepatic immunosuppression. MDSCs can secrete nitric oxide, reactive oxygen species (ROS), and vascular endothelial growth factor (VEGF), thereby promoting Treg recruitment and suppressing cytotoxic T lymphocyte (CTL) activity. Similarly, M2-like TAMs express immunoregulatory molecules such as PD-L1, PD-L2, B7-H4, and VEGF, thereby promoting CTL suppression and functional exhaustion. In addition, Fas-positive CTLs, which normally mediate tumor cell killing, may undergo apoptosis after interacting with Fas ligand (FasL)-expressing macrophages within the liver [50]. Accumulation of these immunosuppressive populations has been associated with poor prognosis in patients with CRLM and reduced responsiveness to ICIs.

### 5.3. Gut–Liver Axis and Immune Reprogramming

Beyond local immune cell recruitment, the gut–liver axis may also contribute to immune reprogramming in CRLM. Because the liver continuously receives microbial products and metabolites from the intestine through the portal vein, hepatic immune cells are exposed to signals that can promote either tolerance or inflammation. Dysbiosis-derived microbial components may modulate Kupffer cell polarization, dendritic cell maturation, myeloid cell recruitment, NK cell activity, and T-cell responses. For example, *Fusobacterium nucleatum* has been reported to promote CRLM by altering gut microbiota composition and regulating the hepatic immune niche [51]. Microbiota-modified bile acids may also suppress CD8^+^ T-cell effector function [36], whereas tumor-resident bacteria may enhance lactate/lactylation-associated macrophage polarization [37]. These findings suggest that the gut–liver axis may link microbial ecology to metabolic adaptation and immune escape. Nevertheless, this field remains at an early stage, and further studies are needed to determine which microbial signatures actively contribute to CRLM progression and which primarily serve as biomarkers of disease state.

### 5.4. NK Cell Dysfunction in the Hepatic Metastatic Niche

Natural killer (NK) cells constitute an innate immune barrier against early metastatic colonization in the liver. Unlike antigen-specific T cells, NK cells can recognize and eliminate transformed cells without prior sensitization, which makes them relevant during the initial phase of metastatic seeding. However, this surveillance function may be progressively weakened during CRLM progression. Harmon et al. reported that liver-resident NK cells were significantly depleted within CRLM tumors and that tumor-conditioned media induced apoptosis of healthy liver-resident NK cells in vitro. Mechanistically, CRLM-derived lactate reduced extracellular and intracellular pH, induced mitochondrial stress, and promoted NK cell apoptosis, suggesting that metabolic remodeling can directly impair innate antitumor immunity [52]. In addition to lactate-mediated suppression, cytokines and metabolites produced by tumor cells, KCs, TAMs, MDSCs, and activated stromal cells may reduce NK cell cytotoxicity, cytokine production, and tumor cell recognition. Therefore, NK cell dysfunction represents an important link between metabolic adaptation and immune escape in CRLM, although whether therapeutic restoration of NK cell activity can improve clinical outcomes remains to be determined.

### 5.5. Dendritic Cell Dysfunction and Impaired Antigen Presentation

Dendritic cells (DCs) bridge innate immune sensing and adaptive T-cell immunity through antigen uptake, processing, and presentation. In CRLM, defective DC recruitment, maturation, or antigen-presenting capacity may weaken tumor-specific T-cell priming and contribute to resistance to immune checkpoint blockade. This issue appears particularly relevant in mismatch repair-proficient (pMMR) CRC, which accounts for most metastatic CRC cases and generally responds poorly to ICIs. Ho et al. showed that orthotopic pMMR CRC liver metastasis models recapitulated clinical resistance to immune checkpoint blockade and displayed a paucity of T cells and DCs, whereas increasing DC abundance with Flt3L improved immune infiltration and sensitized tumors to immune checkpoint blockade [53]. Clinically, DC vaccination after complete resection of colorectal liver metastases has been tested in a randomized phase II setting, supporting the feasibility of DC-based immunotherapy [54]. Nevertheless, whether DC-targeted strategies can reliably improve recurrence-free or overall survival in CRLM remains insufficiently validated. These findings indicate that impaired antigen presentation is not merely a secondary feature of immune suppression but may represent a central barrier to effective antitumor immunity in CRLM.

### 5.6. Adaptive Immune Evasion and the PD-1/PD-L1 Axis

In the hepatic microenvironment, CRC cells upregulate PD-L1 in response to inflammatory cytokines, such as interferon-γ (IFN-γ) and tumor necrosis factor-α (TNF-α), which are predominantly secreted by activated T cells and KCs. Binding of tumor cell PD-L1 to PD-1 on tumor-infiltrating T cells may promote T-cell exhaustion, characterized by impaired proliferation, diminished cytokine secretion, and attenuated cytotoxic activity [55]. In addition to cytokine-mediated induction, PD-L1 expression can be modulated by multiple regulatory layers, including EVs, non-coding RNAs (lncRNAs, miRNAs, and circRNAs), and growth factors such as epidermal growth factor (EGF), TGF-β, and granulocyte-macrophage colony-stimulating factor (GM-CSF) [56,57]. Multiple oncogenic cascades converge to modulate the PD-1/PD-L1 axis, including PI3K/AKT and MAPK, which promote PD-L1 upregulation, and JAK/STAT and NF-κB signaling, which facilitate recruitment of immunosuppressive cells via chemokines [58]. Clinically, elevated levels of circulating EV-associated PD-L1 and soluble PD-L1 in CRC patients correlate with unfavorable clinical prognosis and serve as potential biomarkers for CRLM progression [59]. Therapeutic blockade of the PD-1/PD-L1 axis or upstream regulators, including PI3K/AKT and JAK/STAT signaling, may restore T-cell function and enhance antitumor immunity, thereby supporting combination immunotherapy strategies in CRLM.

Together, these immune alterations establish a liver-specific suppressive niche that promotes immune evasion, sustains metastatic outgrowth, and provides a rationale for combination immunotherapy (Table 1).

## 6. Crosstalk Between Metabolic, Phenotypic, and Immune Reprogramming

Although metabolic adaptation, phenotypic plasticity, and immune evasion are discussed separately above, these processes are closely interconnected within the hepatic metastatic niche [62]. Metabolic remodeling can reshape immune cell function through lactate accumulation, nutrient competition, and lipid metabolic stress [63]. EMT/EMP programs can alter immune checkpoint expression, stromal remodeling, and immune cell infiltration [64]. In addition, shared upstream signals, including TGF-β, hypoxia, HGF/c-MET, PI3K/AKT/mTOR signaling, and inflammatory cytokines, may coordinate metabolic and phenotypic transitions [65]. Thus, CRLM progression may be better interpreted as an integrated adaptive process rather than as the sum of independent reprogramming events. The following sections summarize these major crosstalk mechanisms and highlight areas where evidence remains incomplete.

### 6.1. How Metabolic Reprogramming Promotes Immune Suppression

Metabolic reprogramming can promote immune suppression in CRLM through several interconnected mechanisms. First, enhanced glycolysis can increase lactate production and extracellular acidification. This acidic environment can impair cytotoxic T-cell and NK cell function and may favor M2-like macrophage polarization [52,66]. In CRLM, lactate-mediated acidification has been reported to induce mitochondrial stress and apoptosis in liver-resident NK cells, suggesting a mechanistic link between tumor metabolism and impaired innate immune surveillance. Second, high glucose consumption by tumor cells may create metabolic competition with effector T cells, thereby limiting T-cell glycolysis, mTOR activity, cytokine production, and cytotoxic function [63]. Third, altered lipid metabolism may contribute to immune escape. Fatty acid uptake and oxidation can support tumor cell survival in the lipid-rich hepatic niche, whereas lipid accumulation in dendritic cells can impair antigen presentation and weaken T-cell priming [67]. Finally, glutamine and cholesterol remodeling may further shape the immune microenvironment by supporting tumor growth, myeloid cell recruitment, and stress adaptation. Therefore, metabolic reprogramming should be viewed not only as a tumor-intrinsic survival mechanism but also as a microenvironmental process that may restrict antitumor immunity. However, most evidence linking these metabolic programs to immune suppression in CRLM remains preclinical or translational, and clinical validation is still needed.

### 6.2. How EMT/EMP Alters Immune Checkpoint Expression

EMT and EMP may also promote immune escape by altering immune checkpoint expression and immune cell infiltration. TGF-β-driven EMT can remodel the ECM, activate stromal fibroblasts, and promote T-cell exclusion, thereby limiting direct contact between cytotoxic lymphocytes and tumor cells [64]. In parallel, EMT-associated transcriptional programs may regulate immune checkpoint expression. The ZEB1/miR-200 axis provides one well-described example: loss of miR-200 during EMT can relieve repression of PD-L1, leading to increased PD-L1 expression and suppression of CD8^+^ T-cell activity [68,69]. Other EMT-associated regulators and pathways, including Snail, Twist, PI3K/AKT, MAPK, NF-κB, and JAK/STAT signaling, may also converge on PD-L1 regulation or chemokine secretion, thereby promoting myeloid cell recruitment and T-cell dysfunction [70]. In CRLM, this EMT-immune checkpoint connection is biologically plausible because TGF-β, HGF/c-MET signaling, stromal activation, and immune exclusion frequently coexist in the hepatic metastatic niche. Nevertheless, direct evidence linking specific EMT states to immune checkpoint expression in human CRLM remains limited. Future studies using spatial transcriptomics, multiplex immunofluorescence, and longitudinal sampling will be needed to determine whether EMT precedes immune escape, emerges in response to immune pressure, or evolves together with metabolic adaptation.

### 6.3. Simultaneous Versus Sequential Coupling of EMT and Metabolic Reprogramming

The temporal relationship between EMT and metabolic reprogramming in CRLM is unlikely to follow a single fixed sequence [65,71]. In some settings, these processes may occur simultaneously because they are driven by shared upstream signals, including TGF-β, hypoxia, HGF/c-MET, PI3K/AKT/mTOR signaling, and inflammatory cytokines [65]. These pathways can coordinate EMT-associated transcriptional programs with changes in glycolysis, mitochondrial function, glutamine utilization, lipid uptake, and fatty acid oxidation. In other contexts, metabolic stress may precede and facilitate partial EMT, as glucose deprivation, lactate accumulation, extracellular acidosis, lipid availability, or glutamine dependence may activate stress-responsive pathways that favor hybrid epithelial/mesenchymal states [72]. Conversely, EMT may secondarily increase energy demand and promote mitochondrial plasticity, oxidative-stress tolerance, and dependence on alternative substrates, such as fatty acids or glutamine. Therefore, EMT and metabolic reprogramming should be viewed as dynamically coupled and potentially bidirectional rather than as a linear cascade. However, direct evidence defining the temporal order of these processes in human CRLM remains limited, because many studies rely on endpoint assays, bulk transcriptomics, or in vitro models [62]. Moreover, although this review proposes an integrated model linking metabolic remodeling, phenotypic plasticity, and immune evasion, current evidence is mainly derived from convergent findings across independent studies rather than from a single experimental system demonstrating all three programs simultaneously. Future studies integrating spatial multi-omics, lineage tracing, metabolomics, and multiplex immune profiling in patient-derived CRLM models are needed to clarify when and how these adaptive programs are coordinated during hepatic colonization.

## 7. Therapeutic Vulnerabilities and Future Development

**Figure 2:**
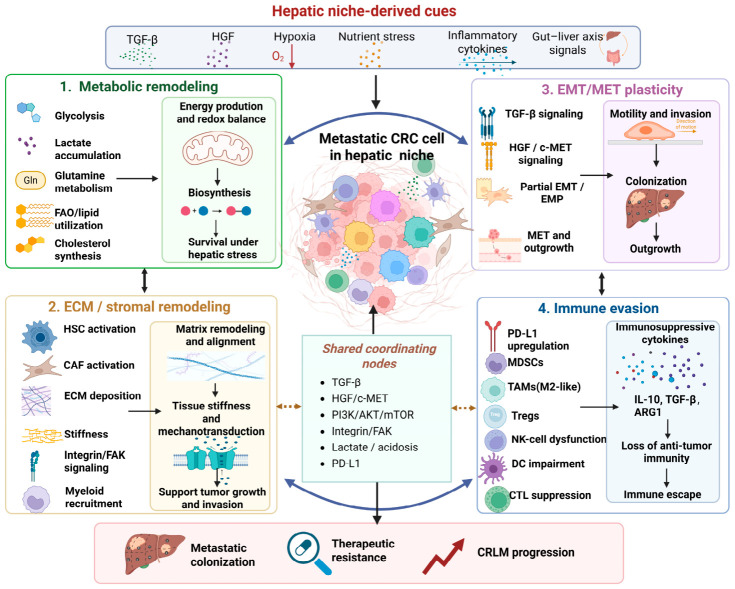
Reciprocal interactions among metabolic remodeling, HSC/CAF-mediated ECM remodeling, EMT/MET plasticity, and immune evasion in colorectal liver metastasis. The hepatic metastatic niche coordinates multiple adaptive programs in CRLM. Hepatic niche-derived cues, including TGF-β, HGF, hypoxia, nutrient stress, inflammatory cytokines, and gut–liver axis signals, converge on shared signaling nodes such as HGF/c-MET, PI3K/AKT/mTOR, integrin/FAK, lactate/acidosis, and PD-L1. These signals promote metabolic remodeling, characterized by enhanced glycolysis, lactate accumulation, glutamine metabolism, fatty acid oxidation, lipid utilization, and cholesterol synthesis. In parallel, activated HSCs and CAFs mediate ECM deposition, tissue stiffness, and integrin/FAK signaling, thereby facilitating EMT/mesenchymal–epithelial transition (MET) plasticity, invasion, stress tolerance, and metastatic outgrowth. EMT/epithelial–mesenchymal plasticity (EMP) can further contribute to immune evasion by promoting PD-L1 expression, cytokine alteration, and immune exclusion. Immune escape is reinforced by MDSCs, TAMs, Tregs, NK cell dysfunction, dendritic cell impairment, and cytotoxic T lymphocyte suppression. These reciprocal interactions collectively promote metastatic colonization, therapeutic resistance, and CRLM progression. Created with BioRender.com.

The three interconnected metabolic, phenotypic, and immune reprogramming axes appear to shape CRLM progression and therapeutic resistance (Figure 2) [62]. However, these mechanisms differ substantially in translational maturity. The metabolic vulnerabilities discussed in this review, including glutamine dependence, fatty acid oxidation, cholesterol synthesis, ectopic gluconeogenesis, and lactate-mediated immune suppression, are supported mainly by preclinical models, patient-derived systems, or translational analyses and have not been established as standard therapeutic targets in CRLM. Similarly, strategies targeting EMT/EMP, such as TGF-β receptor inhibition, integrin blockade, and FAK inhibition, remain largely preclinical or investigational in CRLM. In contrast, immune checkpoint blockade has established clinical benefit in molecularly selected dMMR/MSI-H metastatic CRC, whereas ICI-based combinations for pMMR/MSS CRLM remain under active investigation [73,74,75,76]. This distinction highlights the need to avoid overinterpreting biologically compelling mechanisms before prospective CRLM-specific validation is available. Given the adaptive plasticity of metastatic tumor cells, rational combination strategies may be needed to disrupt multiple metabolic, phenotypic, and immune programs.

### 7.1. Targeting Metabolic Vulnerabilities

CRLM cells may exhibit multiple potentially actionable metabolic dependencies. Inhibition of glutaminase 1 (GLS1) with agents such as CB-839 (telaglenastat) may target glutamine dependence; FAO inhibitors, including etomoxir, may interfere with CPT1A-dependent lipid utilization; mTOR inhibitors such as sirolimus may impair growth programs linked to OXPHOS dependence; and statins may inhibit sterol regulatory element-binding protein 2 (SREBP2)-associated cholesterol biosynthesis [77,78]. In addition, targeting argininosuccinate synthetase 1 (ASS1) to disrupt the urea cycle represents an emerging therapeutic strategy [79,80].

### 7.2. Reversing Phenotypic Plasticity

Emerging evidence suggests that cells in partial EMT states may exhibit distinct metabolic and signaling dependencies. For example, partial EMT cells rely on autophagy- and integrin-mediated signaling for survival, creating opportunities for targeted intervention. Phenotypic plasticity has therefore been proposed as a therapeutic vulnerability, although most supporting evidence remains preclinical. TGF-β receptor inhibitors, such as galunisertib, and agents targeting downstream effectors, including integrins and focal adhesion kinase (FAK), have shown preclinical activity in modulating EMT-associated phenotypes and sensitizing CRLM cells to chemotherapy [81,82]. In addition, targeting fibroblast activation protein-α (FAPα) in inflammatory cancer-associated fibroblasts (iCAFs) may disrupt the CXCL5–CXCR2 paracrine signaling axis that supports mesenchymal phenotypes [83].

### 7.3. Restoring Antitumor Immunity

Although ICIs have limited efficacy as monotherapy in CRLM, combination strategies have shown encouraging therapeutic potential, largely by targeting multiple components of the hepatic immune microenvironment. Preclinical and early translational studies suggest that CSF1R inhibitors may reprogram KCs, whereas CXCR2 inhibition may reduce MDSC and neutrophil recruitment. In parallel, alternative immune checkpoints, such as lymphocyte-activation gene 3 (LAG-3), are being investigated to enhance antitumor immunity. Emerging clinical evidence suggests that selected combinatorial approaches may have activity in defined molecular or clinical subgroups. In the phase II CheckMate-142 trial, nivolumab plus relatlimab was associated with an objective response rate (ORR) of 39% in patients with liver-metastatic mismatch repair-deficient (dMMR)/microsatellite instability-high (MSI-H) CRC [74]. For pMMR/MSS CRC, the CAMILLA trial reported that cabozantinib, a multitarget tyrosine kinase inhibitor targeting VEGFR2, MET, AXL, and related pathways, combined with durvalumab was associated with a 27.6% ORR in liver-metastatic disease [84]. More recently, the phase III STELLAR-303 trial reported an overall survival benefit for zanzalintinib plus atezolizumab versus regorafenib in non-MSI-H/dMMR metastatic CRC; however, liver-metastasis-specific validation remains warranted [75]. Collectively, combination strategies targeting the hepatic microenvironment hold promise for enhancing immunotherapeutic efficacy in CRLM.

### 7.4. Synergistic and Next-Generation Immunotherapies

Crosstalk among the three reprogramming axes provides a mechanistic rationale for multi-targeted therapeutic strategies. Key targeted agents modulating the CRLM microenvironment, currently in preclinical or clinical development, are summarized in Table 2. Potential combinatorial strategies include metabolic inhibitors combined with ICIs (e.g., GLS1 inhibitors plus anti-PD-1), EMT inhibitors combined with ICIs (e.g., galunisertib plus anti-PD-L1), and multitargeted regimens designed to disrupt several reprogramming axes. Beyond conventional combination regimens, next-generation immunotherapeutic modalities are being investigated, including chimeric antigen receptor (CAR)-T cell therapy, bispecific T-cell engagers (BiTEs), tumor-infiltrating lymphocyte (TIL) therapy, and oncolytic virotherapy [85]. These emerging strategies are designed to enhance immune activation and tumor specificity through distinct mechanisms, but their efficacy and safety in CRLM require further validation.

## 8. Challenges and Future Directions

The therapeutic opportunities discussed above arise from hepatic niche-driven metabolic, phenotypic, and immune reprogramming [62]. However, these same adaptive processes also create major barriers to clinical translation. Metabolic plasticity can enable compensatory substrate use, EMT/EMP can generate reversible drug-tolerant states, and immune remodeling can limit the efficacy of checkpoint blockade or cell-based immunotherapy [64]. Therefore, future CRLM therapeutic development requires not only rational drug combinations but also improved biomarkers, more faithful preclinical models, and adaptive strategies that account for spatial and temporal heterogeneity within the liver metastatic niche [100].

Molecular subtype-specific adaptation also requires greater attention [101,102]. Although hepatic niche-derived pressures, such as nutrient limitation, stromal activation, hypoxia, and immune selection, may affect many CRLM lesions, the adaptive programs used by tumor cells are unlikely to be identical across molecular backgrounds. KRAS- or BRAF-mutant tumors may exhibit distinct metabolic, inflammatory, and stromal interaction programs; TP53 alterations may influence stress tolerance and phenotypic plasticity; and APC/Wnt pathway activation has been associated with immune exclusion [103]. In addition, MSI-H/dMMR and pMMR/MSS tumors differ substantially in neoantigen burden, immune infiltration, and response to immune checkpoint blockade [104]. Therefore, metabolic, phenotypic, and immune reprogramming should be interpreted in a subtype-aware manner rather than as universal features of all CRLM. Future preclinical and clinical studies should incorporate molecular stratification to determine which adaptive vulnerabilities are broadly shared and which are restricted to specific genetic or immunological subtypes [105].

These molecular and microenvironmental differences also complicate the clinical translation of microenvironment-targeted therapies. Several barriers currently limit their clinical translation in CRLM [62]. First, treatment-related toxicity remains a major concern. Because the liver is a central metabolic and immune organ, metabolic inhibitors, macrophage-targeting agents, TGF-β pathway inhibitors, and immune checkpoint combinations may cause hepatotoxicity, immune-related adverse events, cytokine-mediated toxicity, or off-tumor effects on normal stromal and immune cells [106]. Second, adaptive resistance may emerge when a single pathway is inhibited. Tumor cells can switch substrate use, activate compensatory survival pathways, adopt partial EMT states, or upregulate alternative immune checkpoints, thereby limiting the durability of treatment response [107]. Third, biomarker-guided patient selection remains insufficiently developed. Markers such as MSI/MMR status, PD-L1 expression, immune cell infiltration, ctDNA dynamics, EV-associated molecules, CAF markers, metabolic signatures, and spatial immune organization may help stratify patients, but few have been prospectively validated for CRLM-specific treatment selection [64]. Finally, interpatient and interlesional heterogeneity complicates therapeutic development, as different liver metastases within the same patient may exhibit distinct metabolic states, stromal composition, immune infiltration, and genetic alterations [12].

Consistent with the niche-driven mechanisms discussed above, microenvironment-derived biomarkers should be interpreted not only as diagnostic markers but also as dynamic readouts of hepatic metabolic, stromal, immune, and gut–liver axis remodeling. Conventional imaging modalities, including CT, MRI, and PET-CT, exhibit limited sensitivity for detecting micrometastases smaller than 1 cm, while classic serum biomarkers such as CEA and CA19-9 lack sufficient specificity and sensitivity for early CRLM detection [108]. Accordingly, circulating and tissue-derived TME components have emerged as candidate markers for liquid biopsy-based surveillance and treatment monitoring. CAF-associated FAP may reflect stromal activation and ECM remodeling [109]. EV-associated molecules, including miR-934, miR-203a-3p, circSATB1, PD-L1, and integrin αvβ5, may capture different aspects of liver colonization, immune suppression, and organotropic metastatic signaling [110]. NET-related components, such as neutrophil elastase, citrullinated histone H3, and myeloperoxidase, may indicate inflammation-associated metastatic risk [111]. ctDNA methylation profiles may provide complementary information on tumor burden and molecular evolution [112]. In addition, gut microbiota-associated signatures, including *Fusobacterium nucleatum*, may reflect gut–liver axis remodeling and could complement EVs, ctDNA, and immune biomarkers for early detection, patient stratification, and dynamic monitoring of CRLM [113,114].

Another major challenge is the limited fidelity of conventional preclinical models. Traditional xenograft models lack a fully functional human immune system and cannot recapitulate the complex hepatic metastatic niche, including resident KCs, HSCs, LSECs, and sinusoidal vascular architecture. Advanced platforms, including humanized mouse models, patient-derived organoids, liver-on-a-chip systems, and spatially resolved patient-derived models, are therefore needed to better evaluate microenvironment-targeted combinations. Meanwhile, treatment sequencing requires careful optimization because concurrent inhibition of metabolic, stromal, and immune pathways may increase toxicity or produce antagonistic effects. Biomarker-guided adaptive trial designs may help define optimal dosing schedules, identify responsive subgroups, and monitor emerging resistance.

Future research should focus on the following perspectives: First, biomarker-driven adaptive clinical trials with real-time tissue profiling, along with spatial-omics and longitudinal biopsies to monitor clonal evolution and immune microenvironment remodeling, are needed. Second, advanced preclinical platforms, including humanized mice, patient-derived organoids, and organ-on-a-chip systems, should be adopted to better recapitulate the human liver metastatic niche. Third, the sequencing of combination therapies requires optimization to minimize antagonism and maximize synergy, and repurposing approved drugs such as statins and metformin could accelerate clinical translation. Finally, integration of liquid biopsy markers, including EVs, ctDNA methylation, NET-related components, and gut microbiota-associated signatures, with spatial immune profiling and metabolic signatures may improve early diagnosis, response prediction, and dynamic monitoring in CRLM.

## 9. Conclusions

The liver microenvironment may promote metabolic, phenotypic, and immunological reprogramming in metastatic CRC cells. These adaptive mechanisms create therapeutic barriers but may also provide actionable vulnerabilities for CRLM intervention. Developing precise therapeutic strategies targeting these core reprogramming processes represents a promising approach to improve treatment efficacy and overcome therapeutic resistance in CRLM.

## Figures and Tables

**Table 1:** Immune reprogramming in colorectal liver metastasis.

Immune Component	Reprogrammed Phenotype in CRLM	Dominant Mediators/Signals	Functional Consequence	Therapeutic Implication	References
Kupffer cells (KCs)	M2-like, immunosuppressive polarization; reduced antigen presentation	IL-10, TGF-β, IL-6; CSF1R-dependent polarization	Suppression of CD8^+^ T-cell activity, promotion of Treg recruitment, support of tumor cell survival	KC reprogramming; CSF1R inhibition	[46,60]
Myeloid-derived suppressor cells (MDSCs)	Recruitment and expansion within the hepatic niche	CCL2, CCL5, CXCL5; NO, ROS, VEGF	CTL suppression, angiogenesis, reinforcement of immune tolerance	Myeloid-targeted combinations; suppressive myeloid cell blockade	[60,61]
Tumor-associated macrophages (TAMs)	M2-like polarization; PD-L1/PD-L2/B7-H4-high state	CCL2/STAT3/TCF4/SPP1 axis; TGF-β; VEGF	CTL exhaustion, ECM remodeling, pro-angiogenic signaling	CSF1R-based macrophage reprogramming; VEGF-based combinations	[22,61]
Regulatory T cells (Tregs)	Expansion/accumulation of suppressive FOXP3+ Tregs	IL-10; monocyte PD-L1 induction; myeloid/stromal cues	Reduced CD8^+^ T-cell infiltration and weakened antitumor immunity	Treg modulation combined with ICIs	[60,61]
CD8^+^ T cells	Exhaustion/functional paralysis	PD-1/PD-L1 signaling; IFN-γ- and TNF-α-driven suppression	Impaired proliferation, cytokine secretion, and cytotoxicity	PD-1/PD-L1 blockade; combinations that remodel the hepatic niche	[60,61]
NK cells	Depletion or functional impairment of liver-resident NK cells	Lactate accumulation, acidosis, mitochondrial stress, TGF-β, IL-10, myeloid-derived suppressive mediators	Reduced innate tumor cell killing and weakened early metastatic surveillance	NK cell restoration strategies; metabolic or myeloid-targeted combinations	[52]
Dendritic cells	Reduced abundance, impaired maturation, and defective antigen presentation	Flt3L-sensitive DC paucity, IL-10, TGF-β, lipid stress, tolerogenic hepatic signals	Impaired T-cell priming and reduced responsiveness to immune checkpoint blockade	DC expansion or vaccination strategies; DC-enhancing combinations with ICIs	[53,54]
Tumor cells/PD-L1 axis	PD-L1 upregulation and immune escape	IFN-γ, TNF-α, EVs, ncRNAs, EGF, TGF-β, GM-CSF; PI3K/AKT, MAPK, JAK/STAT, NF-κB	T-cell exhaustion and reduced response to ICIs	Combination checkpoint blockade plus upstream pathway inhibition	[60,61]

**Table 2:** Targeted therapies for the CRLM microenvironment.

Reprogramming Axis	Key Therapeutic Target	Representative Drugs/Strategies	Clinical Setting	Current Status	Reference
Metabolic reprogramming	GLS1, CPT1A, mTOR, OXPHOS, SREBP2, ASS1	Telaglenastat (CB-839), etomoxir, sirolimus, statins, ASS1-directed arginine-deprivation strategies	Liver-dominant or treatment-resistant CRLM with candidate metabolic biomarkers	Preclinical/repurposing rationale/early clinical exploration	[26,86,87,88,89]
Phenotypic plasticity	TGF-β/SMAD, integrin–FAK, FAPα	Galunisertib, FAK inhibitors, integrin-targeted agents, FAPα-directed stromal therapies	EMT-high or hybrid E/M CRLM; invasion-prone or chemotherapy-resistant lesions; generally explored as combination therapy	Preclinical/early-phase clinical investigation	[83,90,91,92]
Immune reprogramming	PD-1/PD-L1, CTLA-4, LAG-3, CSF1R, CXCR2, VEGF/TAM axis	Pembrolizumab, nivolumab ± ipilimumab, bevacizumab-based combinations, CSF1R inhibitors, CXCR2 antagonists	dMMR/MSI-H CRLM for checkpoint blockade; pMMR/MSS CRLM mainly in biomarker-selected combination or microenvironment-remodeling strategies	Approved for MSI-H/dMMR mCRC; investigational for MSS/pMMR CRLM combinations	[93,94,95,96,97,98,99]

Abbreviations: CRLM, colorectal liver metastasis; EMT, epithelial–mesenchymal transition; E/M, epithelial/mesenchymal hybrid state; iCAF, inflammatory cancer-associated fibroblast; OXPHOS, oxidative phosphorylation; FAK, focal adhesion kinase; TAM, tumor-associated macrophage.

## Data Availability

No new data were created or analyzed in this study. Data sharing is not applicable to this article.

## Abbreviations

The following abbreviations are used in this manuscript:

ASS1	Argininosuccinate synthetase 1
ATP5A	ATP synthase F1 subunit alpha
BiTEs	Bispecific T-cell engagers
CAF	Cancer-associated fibroblast
CAR	Chimeric antigen receptor
CPT1A	Carnitine palmitoyltransferase 1A
CRC	Colorectal cancer
CRLM	Colorectal liver metastasis
CSF1R	Colony-stimulating factor 1 receptor
CTL	Cytotoxic T lymphocyte
CTLA-4	Cytotoxic T-lymphocyte-associated protein 4
CTCs	Circulating tumor cells
ctDNA	Circulating tumor DNA
CYP2E1	Cytochrome P450 2E1
DC	Dendritic cell
dMMR	Mismatch repair-deficient
ECM	Extracellular matrix
EGF	Epidermal growth factor
E/M	Epithelial/mesenchymal
EMP	Epithelial–mesenchymal plasticity
EVs	Extracellular vesicles
FAK	Focal adhesion kinase
FAO	Fatty acid oxidation
FAP	Fibroblast activation protein
FasL	Fas ligand
FOXO1	Forkhead box O1
GLS	Glutaminase
GLS1	Glutaminase 1
GLUT1	Glucose transporter 1
GM-CSF	Granulocyte–macrophage colony-stimulating factor
GSH	Glutathione
HGF	Hepatocyte growth factor
HSCs	Hepatic stellate cells
iCAFs	Inflammatory cancer-associated fibroblasts
ICIs	Immune checkpoint inhibitors
IFN-γ	Interferon-γ
KCs	Kupffer cells
LAG-3	Lymphocyte-activation gene 3
LSECs	Liver sinusoidal endothelial cells
MET	Mesenchymal–epithelial transition
MSI-H	Microsatellite instability-high
NAFLD	Non-alcoholic fatty liver disease
NET	Neutrophil extracellular trap
ORR	Objective response rate
OXPHOS	Oxidative phosphorylation
PCK1	Phosphoenolpyruvate carboxykinase 1
pMMR	Mismatch repair-proficient
ROS	Reactive oxygen species
S100A8	S100 calcium-binding protein A8
SLC1A3	Solute carrier family 1 member 3
SREBP2	Sterol regulatory element-binding protein 2
TAMs	Tumor-associated macrophages
TGF-β	Transforming growth factor-β
TIL	Tumor-infiltrating lymphocyte
TME	Tumor microenvironment
TNF-α	Tumor necrosis factor-α
Treg	Regulatory T cell
USF2	Upstream transcription factor 2
VEGF	Vascular endothelial growth factor
YTHDF1	YTH N6-methyladenosine RNA-binding protein F1
